# Enhancing PLA Strength and Layer Adhesion: Physical and Microstructural Insights from Vibration-Assisted FFF/FDM

**DOI:** 10.3390/polym18141767

**Published:** 2026-07-20

**Authors:** Lotfi Ben Said, Fouzi Alhadar, Hamdi Hentati, Mondher Wali, Badreddine Ayadi, Sattam Alharbi, Muapper Alhadri

**Affiliations:** 1 Department of Mechanical Engineering, College of Engineering, Scientific and Engineering Research Center, University of Ha’il, Ha’il 55473, Saudi Arabia; 2 The Higher Institute for Technical Sciences Tarhuna, Tarhuna, Libya; 3 Laboratory of Mechanics, Modeling and Manufacturing (LA2MP), University of Sfax, Sfax 5080, Tunisia; 4 Higher School of Sciences and Technologies of Hammam Sousse, University of Sousse, Sousse 4011, Tunisia; 5 Laboratory of Energy and Materials (LabEM), University of Sousse, Sousse 4011, Tunisia

**Keywords:** polylactic acid (PLA), fused deposition modeling (FDM), vibration-assisted printing, response surface methodology (RSM), scanning electron microscopy (SEM)

## Abstract

Material Extrusion (MEX), particularly Fused Filament Fabrication (FFF), commercially known as Fused Deposition Modeling (FDM), has become one of the most widely used additive manufacturing technologies for producing polymer components. However, the mechanical performance of printed parts remains limited by weak interlayer bonding and internal porosity. This study investigates the effectiveness of controlled low-frequency bed vibration in improving the physical and mechanical properties of PLA components manufactured by vibration-assisted FFF/FDM. The influence of printing speed, raster angle, and vibration level was experimentally evaluated through tensile, flexural, surface roughness, Fourier Transform Infrared Spectroscopy (FTIR), and Scanning Electron Microscopy (SEM) analyses. Response Surface Methodology (RSM) was employed to optimize the process parameters with respect to tensile strength, yield strength, flexural strength, and surface quality. The results demonstrate that moderate bed vibration (Level 2) provides the best overall performance, improving the mechanical properties by approximately 8–15% compared with conventional printing. SEM observations revealed an approximately 60% reduction in average pore size, together with enhanced filament fusion and interlayer adhesion, while FTIR analysis confirmed that the chemical structure of PLA remained unchanged. These findings demonstrate that controlled mechanical bed vibration is a simple and effective strategy for enhancing the quality, reliability, and structural performance of FFF/FDM-printed PLA components. The proposed approach also provides practical guidelines for optimizing additive manufacturing processes and supports the development of advanced polymer manufacturing capabilities in Hail’s growing industrial sector.

## 1. Introduction

The optimization of manufacturing process parameters plays a fundamental role in improving the mechanical performance, dimensional accuracy, and durability of engineering components [1,2,3]. In modern manufacturing, careful control of processing conditions is essential not only to maximize strength and stiffness but also to minimize defects, delay damage initiation, and enhance the long-term reliability of structural parts [4]. These challenges are particularly important in additive manufacturing (AM), where the layer-by-layer fabrication process strongly influences the final microstructure and mechanical behavior of printed components. Consequently, understanding the physical mechanisms relating process conditions to microstructural evolution and phase morphology has become essential for improving the functional behavior of printed parts. Among the different AM processes, fused deposition modeling (FDM) has attracted considerable attention due to its cost-effectiveness and compatibility with various thermoplastic polymers [5,6], especially in many fields in Hail city’s industrial sector. However, FDM-fabricated components exhibit pronounced heterogeneous microstructures, weak interlayer adhesion, and process-induced porosity, all of which significantly affect their physical and mechanical response [7,8]. Polylactic acid (PLA) has emerged as one of the most widely used thermoplastics in FDM owing to its favorable physical and mechanical properties, good interfacial bonding characteristics, and environmental sustainability [9]. Nevertheless, PLA parts fabricated by FDM exhibit pronounced anisotropy that is governed by interfacial physics rather than bulk properties, as rapid cooling restricts polymer chain diffusion, leading to incomplete interlayer healing and heterogeneous phase morphology [10,11]. Consequently, stiffness, strength, and fracture resistance are primarily controlled by interlayer cohesion and void distribution, which govern damage initiation and propagation under mechanical loading. 

Several studies have shown that inadequate parameter selection can lead to poor layer adhesion, increased defects, and reduced mechanical integrity. Infill density and layer thickness are two critical process parameters that strongly influence the mechanical and physical performance of printed components [12]. Raster angles, which directly influence crack propagation and inter-road bonding, and print speed, which affects interlayer adhesion and void formation, also play a crucial role in defining the mechanical response and structural integrity of printed parts [13]. Wang et al. [14] investigated the influence of process parameters on the dynamic mechanical behavior of FDM-printed PLA, particularly under variable temperature conditions, and examined the mechanisms through which these parameters affect the mechanical response of the printed thermoplastic. To improve microstructural quality and enhance material properties, various strategies have been explored, including fiber reinforcement and process modifications such as vibration-assisted printing. In fact, vibrations in FDM systems can significantly influence deposition accuracy and internal structure. Flexible thermoplastics such as PLA are particularly sensitive to vibration-induced deformation during printing. Controlled mechanical vibrations have been successfully employed in conventional manufacturing processes to enhance material densification, reduce voids, and improve microstructural homogeneity [15,16]. In additive manufacturing, two distinct vibration-assisted approaches have been investigated. High-frequency ultrasonic vibration (typically in the kHz range), applied to the extruder or nozzle, directly interacts with the molten polymer by reducing its apparent viscosity, increasing molecular mobility, enhancing wetting between adjacent filaments, and promoting stronger interlayer diffusion. It has also been shown to improve the surface quality of printed parts by reducing the staircase effect and enabling the deposition of thinner, more uniform layers [17]. Tofangchi et al. [18] reported that applying 34.4 kHz ultrasonic vibration through the print head during ABS FDM improved interlayer adhesion by up to 10%, without altering thermal conditions, due to enhanced chain reptation and entanglement at the interface. Similarly, Wu et al. [19] introduced substrate-based ultrasonic vibration, and in situ IR monitoring revealed localized temperature increases and higher molecular mobility, which promoted faster interlayer diffusion and improved bonding. Maidin et al. [20] found that in FDM-printed ABS and PLA, a *Z*-axis orientation improved compressive strength, while ultrasonic vibration had little effect on strength or bonding. Surface roughness increased at 10 kHz but smoothed at 20 kHz, with the best results achieved with a *Z*-axis orientation and 0 kHz vibration. These findings highlight the significant potential of vibration assistance in improving microstructural integrity and mechanical performance. In contrast, the present work investigates low-frequency mechanical vibration (typically in the Hz range) generated by an eccentric motor and transmitted to the build platform. Unlike ultrasonic excitation, this low-frequency mechanical excitation does not significantly modify the rheological properties of the polymer melt. Instead, it improves filament accommodation and consolidation, facilitates the release of trapped air, enhances interfacial contact between adjacent deposition roads, and consequently reduces porosity while strengthening interlayer bonding.

Since process parameters and dynamic printing conditions significantly influence interlayer bonding and failure mechanisms, systematic parameter optimization is essential to ensure consistent and reliable mechanical properties. Combined with experimental characterization, statistical methods offer a powerful tool for modeling nonlinear interactions between manufacturing process variables and predicting optimal conditions. In this context, Heidari-Rarani et al. [21] applied the design of experiments (DOE) method on PLA specimens to experimentally identify the optimal FDM parameters that maximize physical properties while minimizing printing time. The response surface methodology (RSM) method provided a robust and reliable framework for forming process optimization [22,23]. Beyond forming applications, RSM was shown to be an effective method for optimizing machining parameters and improving surface quality and productivity [24,25]. More recently, it was applied in AM processes in order to optimize printing parameters, enhance physical properties, and improve dimensional accuracy of printed components [26,27].

In this context, the present work aims to enhance the mechanical performance of FDM-printed PLA parts by combining vibration-assisted printing with RSM-based process optimization. The mechanical performance of FDM-printed PLA remains highly sensitive to the combination of printing parameters, while dynamic conditions such as bed vibration can significantly influence interlayer adhesion and overall structural integrity of printed components. The novelty of this work lies in the integration of controlled bed vibration as an additional process parameter in statistical multi-response optimization and comprehensive microstructural characterization. Unlike conventional optimization studies that only consider standard printing parameters, the proposed approach systematically investigates the coupled effects of printing speed, raster angle, and vibration level on the mechanical, physical, and microstructural properties of PLA components. Through experimental characterization and statistical modeling, this work seeks to provide a comprehensive framework for improving the reliability and performance of PLA components fabricated via vibration-assisted FDM. The findings are expected to support the advancement of additive manufacturing capabilities in emerging industrial hubs such as Hail City, where developing local expertise in high-quality polymer processing aligns with the region’s broader industrial diversification goals.

## 2. Materials and Methods

### 2.1. Experimental Setup: Vibration-Assisted FDM Printing System

**Figure 1:**
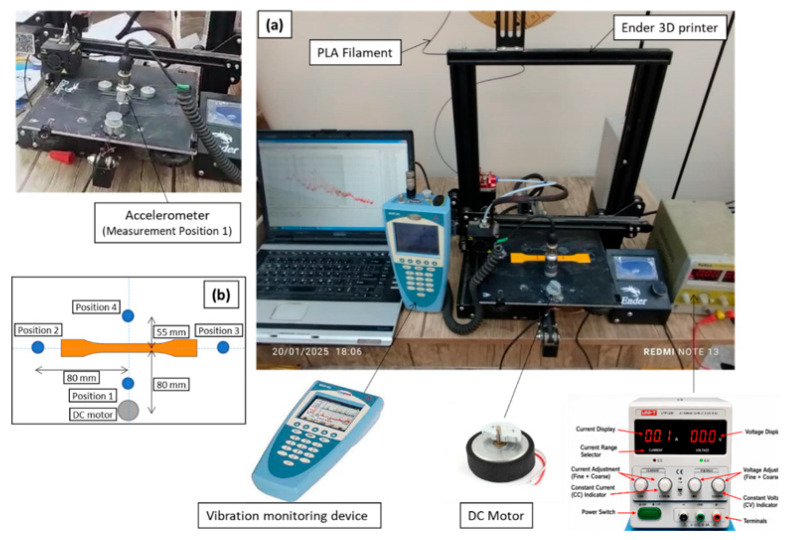
Experimental configuration of the vibration-assisted FDM system: (**a**) modified Creality Ender-3 Pro printer; (**b**) schematic of top view showing the measurement points (P1–P4).

To evaluate the impact of mechanical vibration on the quality of FDM-printed parts, a standard Creality Ender-3 Pro printer (Shenzhen Creality 3D Technology Co., Ltd., Shenzhen, China) was modified to incorporate controlled vibration during the printing process (Figure 1). In this study, EasyFil™ PLA (EPLA-175BLCK-00750, Duiven, The Netherlands) filament with a diameter of 1.75 mm was used for all experiments. All the experiments were designed to align with the existing capabilities of the University of Hail for industrial applications.

While typically considered detrimental in manufacturing, vibrations were intentionally applied in this study to enhance interlayer bonding and improve mechanical properties. A 12 V eccentric rotating mass (ERM) motor was installed beneath the heated build platform to generate mechanical oscillations during printing. The motor was powered by a calibrated DC power supply, enabling precise adjustment of vibration intensity through voltage control. Three voltage levels (2.3 V, 4.6 V, and 6.9 V) were selected to assess the influence of vibration amplitude on the printed material behavior. The motor’s placement was optimized to ensure uniform vibration across the build surface without compromising print stability or dimensional accuracy. As illustrated in Figure 1a, the vibration system was integrated directly into the printer bed, while Figure 1b presents a top-view schematic highlighting the key measurement locations (P1–P4) and placement of the accelerometer used for vibration analysis. Post-printing, specimens were subjected to standardized mechanical tests, including tensile and flexural evaluations under ambient conditions. Surface roughness was also assessed using precision metrology tools. Material properties were calibrated against regionally sourced filament data to improve the study’s relevance to Hail city applications.

### 2.2. Vibration Data Analysis Methods

The objective of the first experiment is to characterize the dynamic behavior of the proposed vibration-assisted FDM printing system and to define the controlled vibration conditions that will be used throughout the experimental study. The vibration characteristics of the 3D printing setup were examined by comparing two operational states: (i) the natural vibrations of the printer during standard operation, and (ii) the modified vibration patterns generated under controlled mechanical excitation. To capture these responses, four positions were strategically chosen at key points on the printer’s frame, especially on the print bed, allowing for a detailed evaluation of vibration propagation across the structure.

**Figure 2:**
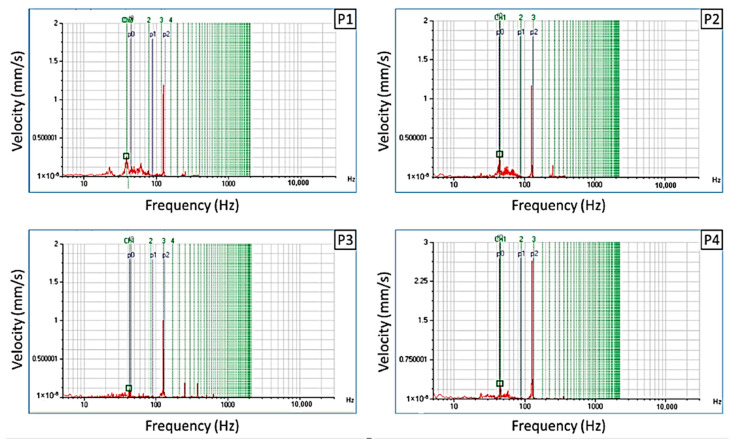
Frequency analysis of the printer at positions surrounding the printed samples.

To characterize the frequency response, Fast Fourier Transform (FFT) analysis was performed on vibration data collected from the print bed. The results, presented in Figure 2, indicate that when the printer operated alone (without external vibration), the most prominent frequency response was around 133.5 Hz. This peak is associated with the inherent oscillations of stepper motors and other moving mechanical elements.

Table 1 summarizes the measured vibration velocities at the four monitored points of the printer during the FDM process. The results demonstrate non-uniform vibration distributions: the highest amplitude was recorded at point P4 (3.59 mm/s), while the lowest occurred at point P3 (2.08 mm/s). These variations highlight localized differences in structural stiffness and load transmission across the build platform, confirming that the vibration effects vary spatially.

The DC motor selected to generate controlled excitation during the FDM process was evaluated through a detailed investigation of its dynamic characteristics. Three input levels (low, medium, and high) were tested to verify that the motor could provide stable and repeatable vibrations without disturbing the operational reliability of the printer. 

**Figure 3:**
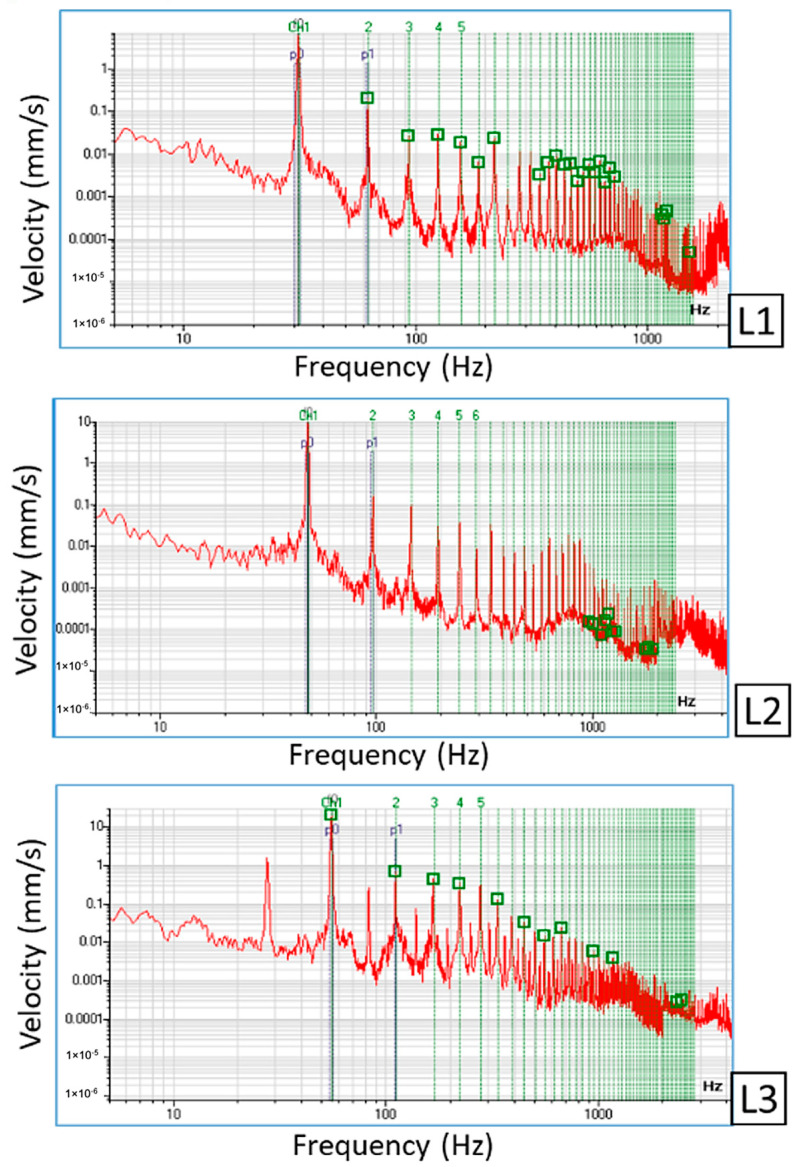
Logarithmic frequency analysis of the DC motor measured near the fixed motor at three rotation levels.

To characterize the dynamic behavior of the vibration-assisted FDM system, the build platform was instrumented with a vibration analyzer to measure the root mean square (RMS) vibration velocity, while the frequency content of the excitation was evaluated using Fast Fourier Transform (FFT) analysis. Figure 3 presents the logarithmic FFT spectra of the DC motor measured near its mounting location for the three motor rotation levels (L1–L3). The corresponding vibration characteristics are summarized in Table 2, including the RMS vibration velocity, the amplitudes of the fundamental frequency (1 × Fo) and its second harmonic (2 × Fo), together with the motor rotational speed and excitation frequency. The RMS vibration velocity increased from 6.88 mm/s at Level 1 to 29.3 mm/s at Level 3, reflecting the increase in excitation intensity. The FFT spectra show that the vibration response is dominated by the fundamental rotational frequency (1 × Fo), while the second harmonic (2 × Fo) remains comparatively small, indicating stable motor operation with no significant dynamic anomalies such as severe rotor imbalance or resonance. Overall, the results validate the suitability of the chosen motor for integration into the modified FDM system, as it can deliver precise vibration input without impairing print accuracy.

### 2.3. Vibration Parameter Selection

In this study, mechanical vibration was introduced as an additional design factor to evaluate its effect on the mechanical properties of PLA parts fabricated by FDM. As summarized in Table 3, four controlled vibration conditions were defined, ranging from standard printing without vibration (baseline) to the maximum stable vibration level before noticeable quality degradation. The highest level was selected as the level just below the threshold causing dimensional defects or weak interlayer bonding. A dedicated DC motor mounted on the printer bed and monitored with a vibration analyzer ensured accurate control and reproducibility of the vibration parameters during the experiments.

**Figure 4:**
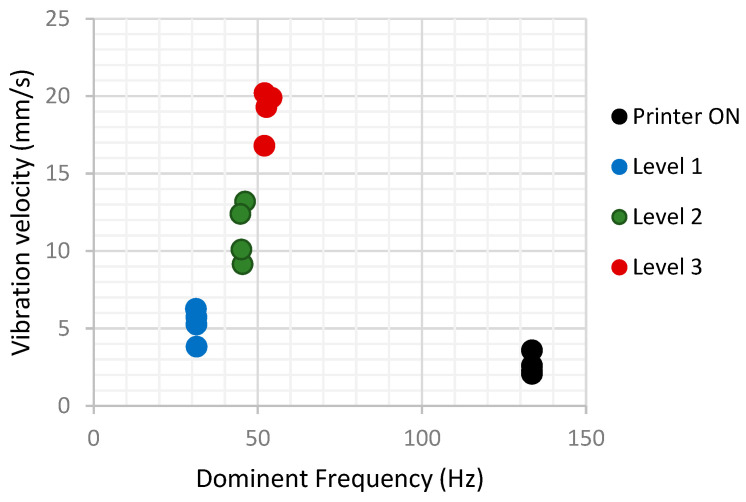
Velocity magnitude measured at four monitoring points.

Figure 4 illustrates the relationship between vibration frequency and velocity across the four measurement positions under all test conditions. The data revealed that the dominant frequency remained constant across the print bed for each vibration level, while amplitude varied depending on the monitoring point, showing how vibration energy was distributed spatially. The reference case (Printer ON) was characterized by a high-frequency response (~133.5 Hz) with low amplitude (~2.63 mm/s). In contrast, vibration-assisted levels exhibited lower dominant frequencies (31–53 Hz) with higher amplitudes (~5.27 to ~19.05 mm/s), confirming that the DC motor introduced controlled excitation distinct from the printer’s inherent vibrations. In addition, experimental observations demonstrated a strong dependency of vibration velocity on the DC motor’s rotational speed (rpm), confirming that motor speed governs the system’s dynamic response. At Level 1, vibration velocity values ranged between 3.83 mm/s (P4) and 6.27 mm/s (P1), averaging around 5.27 mm/s. This is significantly higher than the baseline case, where the printer operating alone (Printer ON) produced an average of 2.63 mm/s, highlighting the capability of the vibration-assisted setup to provide additional mechanical energy that can enhance interlayer adhesion and reduce void formation.

### 2.4. Printing Parameters

In this study, two specimen groups were fabricated. The first group (five samples) was printed under standard FDM conditions without vibration and used as a reference. The second group consisted of three subgroups, each subjected to a different controlled vibration level during printing, with five specimens per condition. Standardized specimen geometries were adopted for all mechanical characterizations. Tensile specimens were fabricated in accordance with ISO 527-2 Type B [28] and used to determine the tensile strength and yield strength. Flexural specimens were prepared following ISO 178 [29] for three-point bending tests. All other printing parameters (nozzle temperature, bed temperature, layer thickness, etc.) were kept constant to ensure that any differences in mechanical response were solely due to vibration (Table 4).

## 3. Experimental Results and Discussions

**Figure 5:**
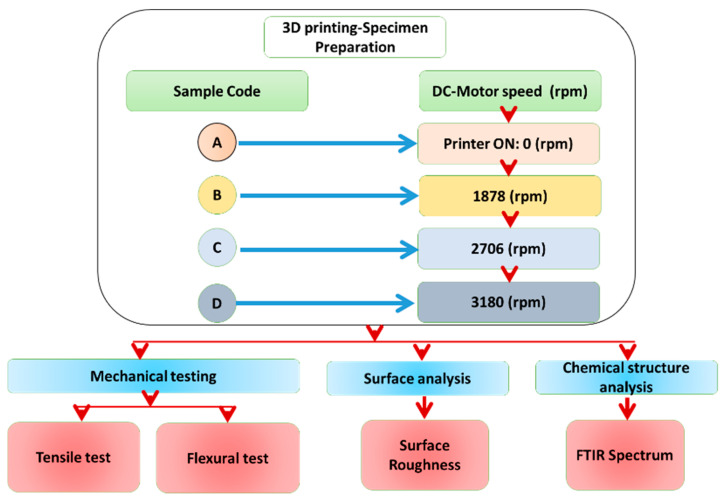
Methodology flowchart for characterization of PLA manufactured by vibration-assisted FDM.

The study framework includes chemical structure analysis, mechanical testing (tensile and flexural), dimensional analysis, and surface characterization, as illustrated in Figure 5. A statistical analysis was conducted to compare the mechanical properties of vibrated and non-vibrated samples. 

### 3.1. Chemical Structure Analysis

Chemical structure characterization was conducted using Fourier Transform Infrared Spectroscopy (FTIR) with an Agilent Cary 630 FTIR spectrometer equipped with an Attenuated Total Reflection (ATR) accessory (Agilent Technologies, Santa Clara, CA, USA). For each vibration condition (Levels A–D), small sections were cut from the printed specimens for analysis. FTIR spectra were collected over the range of 400–4000 cm^−1^ with a spectral resolution of 4 cm^−1^, using step-scan mode with four scans per measurement. The FTIR analysis aimed to verify the chemical stability of the PLA polymer under varying vibration levels. This characterization was essential to ensure that the observed improvements in mechanical properties were due to physical effects, such as enhanced interlayer bonding and reduced void content, rather than chemical alterations induced by mechanical vibration.

**Figure 6:**
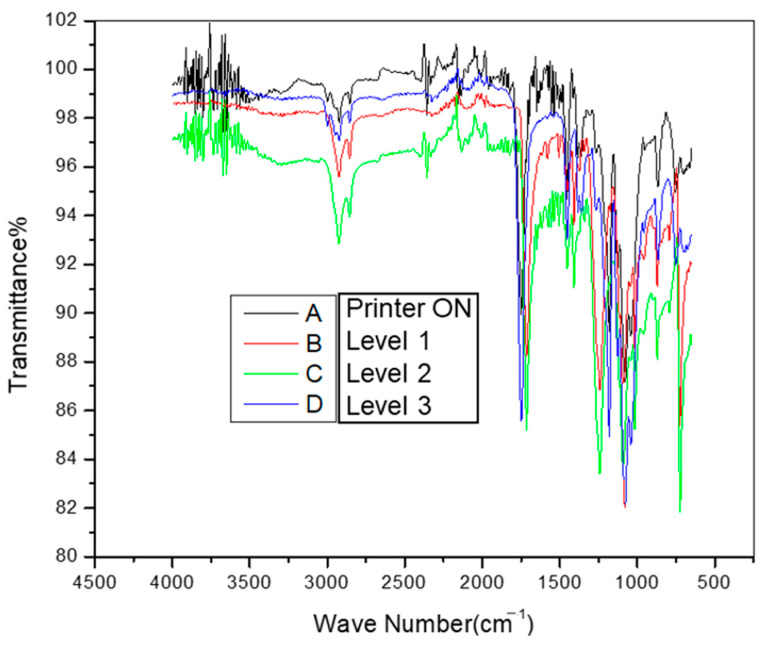
FTIR Spectrum for samples after 3D printing with vibration and without vibration (printing direction: 0°; print speed: 45 mm/s).

The FTIR spectra analysis of the vibration-assisted FDM-printed PLA samples reveals significant insights into the effects of mechanical vibration on material structure (see Figure 6). The control sample (A, no vibration) and vibration-assisted FDM samples (B-Level 1, C-Level 2, D-Level 3) all maintain characteristic PLA peaks, including the peaks for C=O stretch (~1750 cm^−1^) and C-O-C linkage (~1080–1100 cm^−1^), confirming that the printing process preserves the polymer’s fundamental chemistry regardless of vibration conditions. However, notable differences emerged in peak characteristics: Samples B and C (Levels 1–2 vibration) show slightly enhanced transmittance and sharper peaks, suggesting improved molecular alignment and crystallinity due to vibration-induced chain rearrangement. In contrast, Sample D (Level 3) exhibits broader peaks and reduced transmittance, indicating potential disruption of crystalline domains from excessive vibrational energy. The absence of new absorption bands confirms that no chemical degradation or moisture absorption occurred during printing. 

These findings demonstrate that controlled vibration (Levels 1–2) can optimize PLA’s microstructure without compromising chemical integrity, while excessive vibration may introduce structural disorder, highlighting the importance of vibration parameter optimization in additive manufacturing. 

### 3.2. Example of Results from Vibration-Assisted FDM

**Figure 7:**
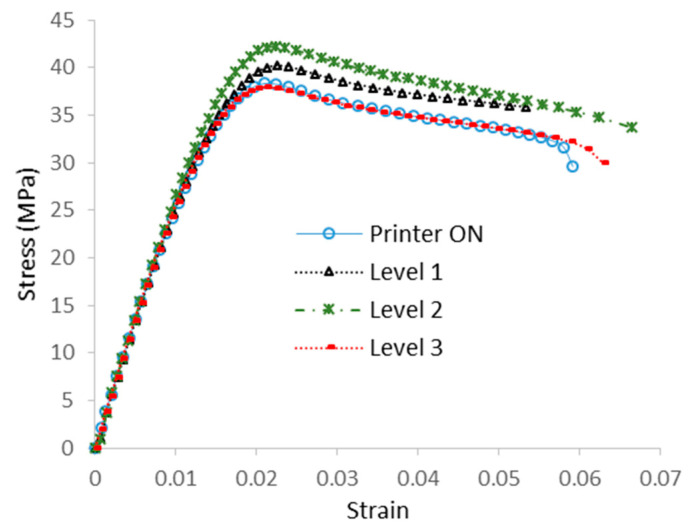
Stress–strain curve of PLA samples printed at different vibration levels (printing direction: 0°; print speed: 45 mm/s).

To evaluate the mechanical properties, tensile tests were conducted using a Universal Testing Machine. Standard specimens were loaded at a constant crosshead speed of 5 mm/min until failure. Both reference PLA samples (without vibration) and vibration-assisted FDM samples were tested. The stress–strain curves (Figure 7) show similar slopes in the elastic region for all conditions, indicating that the applied vibration did not have a significant effect on the Young’s modulus of PLA.

The findings show that applying moderate controlled vibration during printing improves the tensile strength of PLA parts. Compared with non-vibrated specimens, UTS increased by 4.8% and 10.25% at vibration Levels 1 and 2, respectively, due to enhanced interlayer bonding and reduced porosity. In contrast, excessive vibration (Level 3) led to a reduction in UTS, which fell slightly below the reference value, likely as a result of printing defects and induced internal stresses. The elastic modulus remained unchanged at 2.57 GPa for all conditions, indicating that vibration mainly influences strength rather than stiffness.

**Figure 8:**
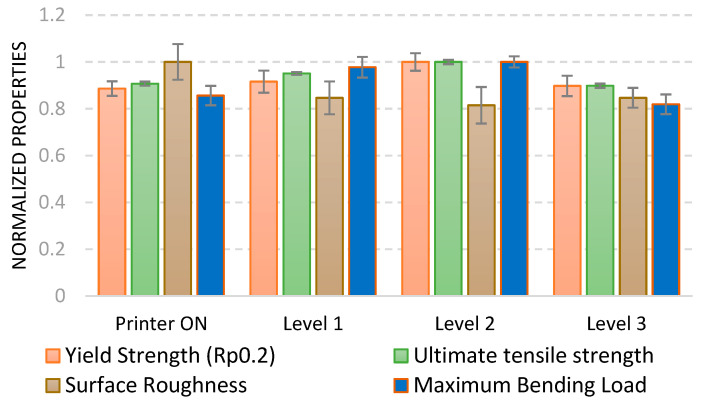
Influence of controlled vibration on FDM test results (printing direction: 0°; print speed: 45 mm/s).

Figure 8 presents a comprehensive overview of the experimental results obtained for PLA specimens manufactured by FDM under varying vibration levels. It illustrates the corresponding surface quality, along with the measured bending loads and tensile properties. This comparative analysis clearly demonstrates how applied vibration influences not only the mechanical performance, particularly in bending and tension, but also the surface finish of the printed parts.

Compared to the non-vibrated condition (Printer ON), moderate vibration (Level 2) delivers the best results. Yield strength (Rp0.2) increases by about 8–12% (≈4–6% over Level 1), ultimate tensile strength increases by ~10% (≈5% over Level 1) and maximum bending load increases by 12–15% (≈6–8% over Level 1). These improvements indicate stronger interlayer bonding and better energy absorption. Overall, Level 2 provides the best balance between improved mechanical performance and acceptable surface finish.

**Figure 9:**
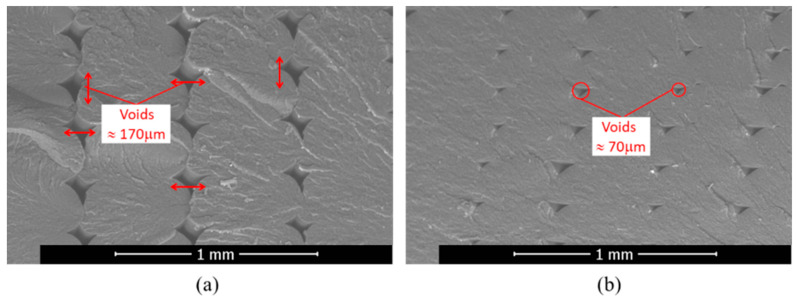
SEM micrographs of PLA manufactured (**a**) without vibration and (**b**) with assisted vibration (Level 2).

Furthermore, microscopic examination of the 3D-printed PLA specimens (Figure 9) highlights the role of vibration-assisted printing in minimizing porosity and improving interline and interlayer bonding.

Without vibration, the printed parts show relatively large pores (0.16–0.17 mm). Applying controlled vibration at Level 2 drastically reduces the pore size to about 6–7.5 μm, corresponding to an average void reduction of 60%. This strong decrease in porosity indicates improved filament fusion and material densification, as confirmed by SEM observations showing a higher density and nearly undetectable interfacial boundaries. Consequently, vibration-assisted FDM at Level 2 significantly enhances the tensile performance of PLA, yielding a more homogeneous microstructure and quasi-isotropic macroscopic mechanical behavior.

### 3.3. Response Surface Methodology (RSM) Analysis

The mechanical and surface properties evaluated in this study are ultimate tensile strength (UTS), yield strength (YS), surface roughness (Ra) and bending load (Fb). A full factorial design was used to examine the combined effects of printing speed (45 and 60 mm/s), raster angle (0° and 90°), and bed vibration (Printer ON, Levels 1–3). The experimental results using different input parameters are presented in Table 5. 

The optimization was based on the Response Surface Methodology (RSM), which uses statistical and mathematical models to describe the relationship between process parameters and mechanical responses. RSM generates regression models and 3D response surface plots to identify the optimal combination of parameters that maximize mechanical performance. The presented surface plots illustrate the individual and interactive effects of printing speed (Sp), raster angle (θ), and vibration amplitude on the mechanical performance and surface roughness of PLA specimens.

**Figure 10:**
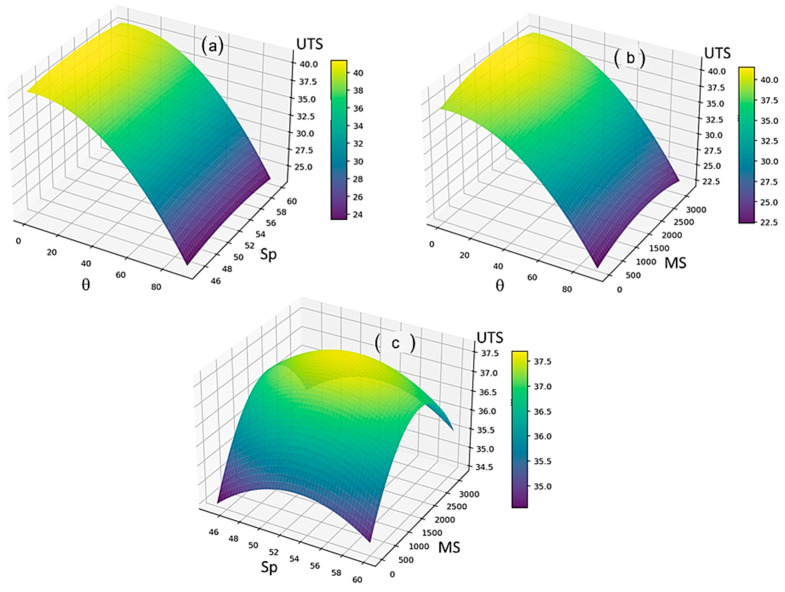
RSM for UTS: (**a**): MS = 2292 rpm; (**b**) Sp = 52.5 mm/s; (**c**) θ = 45°.

In Figure 10, the first surface plot (top left) shows the interaction between raster angle and printing speed. UTS clearly decreases as the raster angle increases from 0° to 90°, regardless of speed. Printing speed has a slight negative effect, since higher speed (60 mm/s) reduces fusion time between layers. The second plot (top right) presents the combined effect of raster angle and vibration amplitude, confirming that both parameters influence UTS. The third plot (bottom) illustrates the interaction between printing speed and vibration amplitude.

The RSM analysis indicates that raster angle is the dominant factor affecting UTS. Specimens printed at 0° show the highest UTS values (up to about 37 MPa), while those printed at 90° exhibit much lower values (often below 25 MPa), highlighting the importance of filament alignment with the tensile load. A lower printing speed (45 mm/s) slightly improves UTS due to better interlayer bonding. Vibration shows a nonlinear effect: moderate amplitude (~2700 rpm) slightly enhances UTS, whereas excessive vibration (~3180 rpm) decreases performance due to deposition instability. The response surfaces clearly confirm these trends and the strong interaction between raster angle and the other parameters.

**Figure 11:**
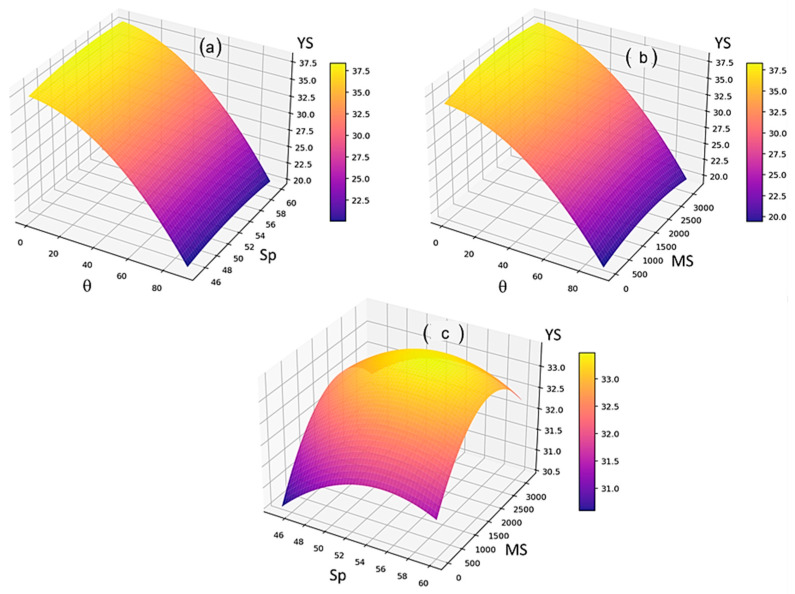
RSM for YS: (**a**): MS = 2292 rpm; (**b**) Sp = 52.5 mm/s; (**c**) θ = 45°.

In addition, Figure 11 presents three response surface plots showing the interactive effects of printing speed (Sp), raster angle (θ), and motor speed (MS), which governs the bed vibration level, on the YS of PLA specimens fabricated via FDM. These visualizations provide insight into how each pair of parameters influences the mechanical behavior of the printed part under static tensile loading.

In the top-left plot, which explores the interaction between θ and Sp, YS is observed to decrease sharply as θ increases from 0° to 90°. This trend confirms that aligning the deposited paths with the tensile load direction (θ ≈ 0°) significantly enhances the material’s yield behavior. Temporarily, Sp shows a weaker effect, with a slight decrease in YS as speed increases from 45 mm/s to 60 mm/s. The top-right plot, which shows the interaction between θ and MS of the vibrated motor, similarly indicates a decline in YS with increasing θ. The impact of vibration appears more moderate. The bottom plot investigates the combined effect of Sp and MS on YS. Similar to UTS, θ exerts the most significant influence on YS. The RSM plots show that YS values for specimens printed at 0° are nearly twice as high as those printed at 90°, with the maximum predicted values reaching over 40 MPa compared to around 21 MPa at 90°. Vibration levels affect the response in a nonlinear but still relevant way: a moderate level (~2700 rpm) slightly improves YS across all settings, likely due to enhanced layer bonding, but higher vibration (~3180 rpm) reduces YS, particularly at 90°, indicating process instability. The effect of Sp is less pronounced but still relevant; increasing the speed from 45 to 60 mm/s generally leads to slight improvements in YS, especially when paired with θ = 0°. These RSM surfaces highlight the secondary effects of interactions that must be accounted for when optimizing FDM parameters.

**Figure 12:**
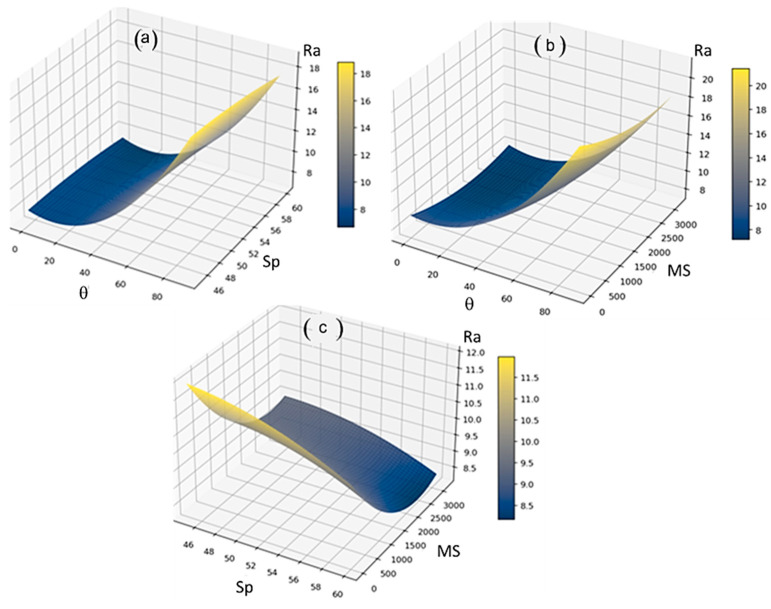
RSM for Ra: (**a**): MS = 2292 rpm; (**b**) Sp = 52.5 mm/s; (**c**) θ = 45°.

In the same context, Figure 12 presents three response surface plots showing the interactive effects of factors on the Ra of PLA specimens printed via FDM. 

In the top-left plot, which illustrates the interaction between θ and Sp, Ra increases significantly as the θ rises from 0° to 90°, highlighting the rougher surface finish associated with perpendicular filament deposition. Printing speed shows a relatively minor effect, with slightly lower Ra values at higher speeds, particularly at low raster angles. In the top-right plot, which explores the interaction between θ and MS, a similar result is observed: surface roughness notably increases with θ, while vibration has a less pronounced but slightly smoothing effect. The bottom plot showing the interaction between printing speed and vibration shows a slight decrease in Ra with increasing vibration, particularly at higher speeds. Overall, the raster angle remains the most dominant factor affecting surface roughness, with lower angles and moderate vibration levels contributing to better surface quality.

**Figure 13:**
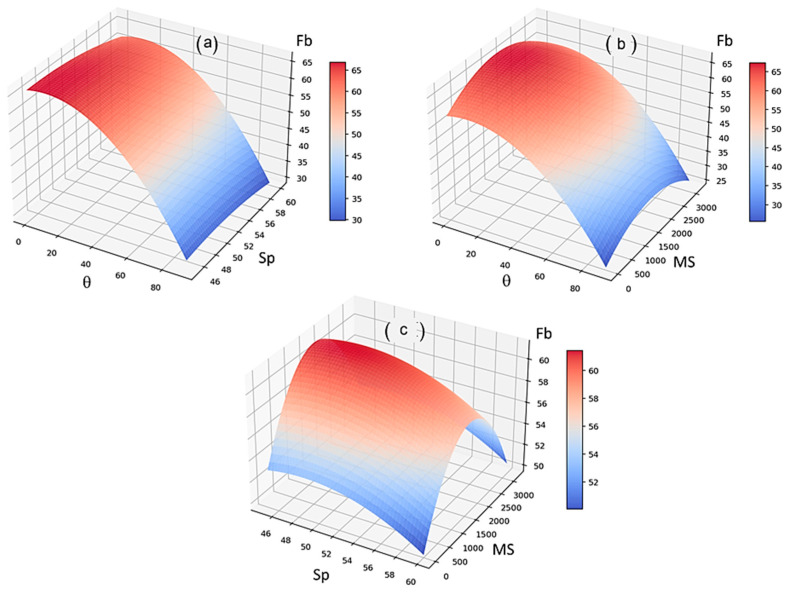
RSM for Fb: (**a**): MS = 2292 rpm; (**b**) Sp = 52.5 mm/s; (**c**) θ = 45°.

Figure 13 presents response surface plots showing the effects of the three factors on the Fb of FDM-printed PLA parts.

Analysis of the RSM surface plots indicates that Fb is mainly influenced by θ. In fact, higher loads were observed at θ = 0° and lower values at θ = 90°. At 0°, Fb reaches the peak value of 68.69 N. The effect of vibration level is also noticeable: moderate levels (around 2700 rpm) enhance Fb, likely by improving material flow and fusion during deposition. However, at the highest vibration (~3180 rpm), a decline in Fb is observed, suggesting that excessive movement destabilizes layer placement. Sp shows a milder influence, with a general tendency for slightly higher Fb at 45 mm/s compared to 60 mm/s, though this effect is less pronounced than those of θ and vibration amplitude (MS). 

### 3.4. Desirability Function Analysis

Desirability function analysis was conducted to determine the optimal combination of process parameters that produced the best overall performance for the printed specimens. The optimal combination of process parameters should simultaneously maximize mechanical performance and minimize surface roughness. The optimization procedure aimed to enhance tensile properties while improving surface quality, ensuring a balanced and efficient manufacturing condition. To achieve this compromise, the desirability function approach was employed. This method transforms each individual response into a dimensionless desirability value ranging from 0 (completely undesirable) to 1 (fully desirable). The overall desirability *D* is then calculated using the geometric mean of the individual desirability values, which is expressed by the following equation:

(1) $$D\left(Y\right)={\left(d_{1}\times d_{2}\times \ldots \times d_{n}\right)}^{1/n}$$

where *d_i_* represents the desirability of each response and *n* is the total number of responses considered.

To perform multi-objective optimization, the desirability function approach was adopted to simultaneously maximize the mechanical properties (UTS, YS, and Fb) and minimize surface roughness (Ra). This method transforms each predicted response obtained from the RSM model into an individual desirability value *d_i_* ranging from 0 to 1. For responses to be maximized (UTS, YS, and Fb), the desirability function, defined by system 1, increases progressively.

(2) $$d_{i}=\left\{\begin{array}{ccc} 0 & & y_{i}\leq L_{i}\\ \left(\frac{y_{i}-L_{i}}{U_{i}-L_{i}}\right) & & L_{i}<y_{i}<\;U_{i}\\ 1 & & y_{i}\geq U_{i} \end{array}\right.$$

where *y_i_* is the response predicted by RSM, *L_i_* is the minimum acceptable limit, and *U_i_* is the target or upper limit. 

Conversely, for the response to be minimized (for surface roughness Ra), the desirability (Equation (3)) decreases gradually as the response approaches the upper acceptable limit and becomes 0 when this limit is exceeded.

(3) $$d_{Ra}=\left\{\begin{array}{ccc} 1 & & y_{i}\leq L_{i}\\ \left(\frac{U_{i}-y_{i}}{U_{i}-L_{i}}\right) & & L_{i}<y_{i}<\;U_{i}\\ 0 & & y_{i}\geq U_{i} \end{array}\;\right.$$

where *y_i_* is the predicted response value, *L_i_* is the ideal minimum value, and *U_i_* is the maximum acceptable value. 

The overall desirability *D* is calculated as the geometric mean of the individual desirability values:

(4) $$D={\left(d_{UTS}\times d_{YS}\times d_{Fb}\times d_{Ra}\right)}^{1/4}$$

The corresponding optimal input parameters required to achieve the optimal global performance are illustrated on Table 6. These results confirm the effectiveness of the RSM-based multi-objective optimization in identifying a robust parameter combination capable of significantly improving both structural performance and surface finish. The high global desirability value (*D* = 0.957 close to 1) indicates an excellent compromise between mechanical performance and surface quality.

## 4. Conclusions

This study demonstrated that the introduction of controlled bed vibration during the FDM process provides an effective means of improving both the mechanical performance and microstructural quality of PLA components. The combined experimental characterization and statistical optimization revealed that vibration level is a critical process parameter that influences interlayer bonding, porosity, and ultimately the structural integrity of printed parts. Among the investigated conditions, Level 2 vibration produced the optimum performance, resulting in improvements of approximately 8–15% in tensile strength, yield strength and flexural strength compared with conventional FDM printing.

Microstructural observations provided clear evidence of the mechanisms responsible for these improvements. SEM analyses showed that controlled vibration promoted better filament consolidation and significantly smaller internal voids, with the characteristic pore size decreasing from approximately 0.16–0.17 mm in conventionally printed specimens to about 0.06–0.07 mm (60–70 μm) in parts printed under the optimal vibration condition, corresponding to an average porosity reduction of nearly 60%. The resulting denser and more homogeneous microstructure enhanced interlayer adhesion and improved load transfer between adjacent filaments, leading to more uniform mechanical behavior. Furthermore, FTIR analysis confirmed that the chemical structure of PLA remained unchanged after vibration-assisted printing, indicating that the observed improvements arose from physical and microstructural modifications rather than chemical degradation of the polymer.

The Response Surface Methodology (RSM) successfully quantified the interactions between printing speed, raster angle, and vibration level, providing predictive models capable of identifying the optimal processing window for maximizing mechanical performance while maintaining good surface quality. The statistical analysis demonstrated that vibration should be carefully controlled since excessive excitation may introduce structural instability, increased surface roughness, and deterioration of mechanical properties. Consequently, controlled vibration should be regarded as an optimization parameter rather than simply using the highest vibration intensity.

Overall, the proposed vibration-assisted FDM approach offers a practical and cost-effective strategy for improving the quality and reliability of PLA components without requiring modifications to the material formulation. The findings provide valuable guidelines for optimizing FDM process parameters and contribute to a better understanding of the relationship between process conditions, microstructure, and mechanical performance. These findings are particularly relevant to the advancement of additive manufacturing in Hail’s growing industrial sector, where enhanced polymer processing capabilities can support the production of high-performance engineering components and promote the adoption of advanced, sustainable manufacturing technologies. 

## Figures and Tables

**Table 1:** Measured vibration amplitudes at four excitation points on the printer during the FDM process.

	P1	P2	P3	P4	Average
Vibration Velocity (mm/s)	2.60	2.26	2.08	3.59	2.63

**Table 2:** Summary of vibration characteristics and motor parameters at three excitation levels.

Parameter	L1	L2	L3
RMS Vibration Velocity (mm/s)	6.88	13.4	29.3
1 × Fo (mm/s)	6.30	9.78	20.3
2 × Fo (mm/s)	0.199	0.17	0.70
Motor Speed (rpm)	1870	2887	3334
Frequency of Motor (Hz)	31.17	48.12	55.57

**Table 3:** Selected DC motor control parameters.

Selected Vibration Level	Sample Code	Voltage (V)	Current (A)	Motor Speed (MS) (rpm)
Printer ON	A	0	0	0
Level 1	B	2.3	0.02	1878
Level 2	C	4.6	0.04	2706
Level 3	D	6.9	0.06	3180

**Table 4:** 3D printing parameters.

Printer Setting	Value
Infill density	100%
Printing temperature	220 °C
Build plate temperature	60 °C
Print speed	45–60 mm/s
Fan speed	100 mm/s
Layer height (mm)	0.2 mm
Printing direction	0–90°

**Table 5:** Experimental results and input parameters.

θ (°)	Sp (mm/s)	Motor Speed (MS) (rpm)	UTS (MPa)	YS (MPa)	Ra (μm)	Fb (N)
0	45	0	38.33	35.05	9.46	58.82
0	45	1878	40.18	36.22	8.01	67.12
0	45	2706	42.26	39.55	7.71	68.69
0	45	3180	37.96	35.5	8.01	56.27
0	60	0	38.08	36.12	8.51	54.12
0	60	1878	39.92	37.05	7.3	61.78
0	60	2706	42.1	40.18	7	63.2
0	60	3180	37.7	35.87	7.4	51.8
90	45	0	21.03	18.23	22.7	24.6
90	45	1878	21.9	18.9	19.2	29.2
90	45	2706	23.8	20.6	18.57	30.2
90	45	3180	21.5	18.47	19.24	25
90	60	0	21.75	18.8	20.45	23.02
90	60	1878	22.48	19.3	17.7	27.24
90	60	2706	24.18	20.92	16.87	28.25
90	60	3180	21.6	18.65	17.53	23.38

**Table 6:** Optimal process parameters.

Category	Parameter/Response	Optimal Value	Unit
Process Parameters	Printing direction (θ)	0	°
	Speed (Sp)	50.3	mm/s
	Motor speed (MS)	2517.2	rpm
Predicted Responses	Ultimate tensile strength (UTS)	41.39	MPa
	Yield strength (YS)	37.87	MPa
	Bending force (Fb)	67.72	N
	Surface roughness (Ra)	7.72	μm
Global Performance	Global desirability (D)	0.942	—

## Data Availability

The original contributions presented in this study are included in the article. Further inquiries can be directed to the corresponding author.
